# Radial Head Osteochondral Flake Refixation With a Transosseous All-Suture Technique

**DOI:** 10.1016/j.eats.2024.103374

**Published:** 2024-12-12

**Authors:** Armin Runer, Marvin Sven Berger, Jose Fernando Sanchez Carbonel, Lucca Lacheta, Julian Mehl, Sebastian Siebenlist

**Affiliations:** a Department of Sports Orthopaedics, Technical University of Munich, Munich, Germany; b University Center of Orthopedics, Trauma and Plastic Surgery, University Hospital Carl Gustav Carus Dresden, Technische Universität Dresden, Dresden, Germany

## Abstract

Elbow dislocations frequently coincide with osteochondral radial head flake fractures, posing a notable risk for subsequent post-traumatic osteoarthritis. While several surgical techniques exist for the reattachment of osteochondral flakes, they often present drawbacks. This article presents a stable fixation of radial head osteochondral fractures using resorbable sutures only. The surgical procedure entails the creation of drill holes through the fragment and radial head, subsequently securing the fragment in place with absorbable sutures in a U-shaped technique. Compared to conventional methods using screws, the presented technique eliminates the need for subsequent implant removal and is economically viable. In summary, this minimally invasive and fragment-preserving approach yields promising clinical outcomes and represents an effective treatment modality for radial head osteochondral fractures in the orthopaedic setting.

Elbow dislocation is the second most common joint dislocation in adults, with an incidence of 5 to 6 per 100,000 inhabitants.^1^ Chondral or osteochondral lesions are reported to be associated in 48.8% of the cases after elbow dislocation.^2^ The most commonly identified lesion sites are the posterolateral capitellum, posterior trochlea, and radial head.^2^ Chondral and osteochondral lesions are associated with an increased risk of post-traumatic pain, leading to early-onset osteoarthritis. The presence of an unstable fragment warrants surgical intervention. Depending on the size of the defect and the integrity of the cartilage, resection or refixation can be performed. Surgical options for smaller lesions are either chondral debridement or bone marrow stimulation. Stable fragment refixation is recommended for larger defects (>1 cm^2^) with good cartilage integrity. Various techniques for osteochondral flake refixation are described, including screws,^3^ flexible wires,^4^ or hydroxyapatite screws. Downsides of the abovementioned refixation methods are the necessity of screw removal or the potential risk of osteolysis. Recently, an easy and cost-effective surgical technique for osteochondral flake refixation using reabsorbable sutures has been described for the knee joint.^5^

In the following, we present a transosseous all-suture technique for stable refixation of an osteochondral radial head flake fracture using resorbable sutures (Video 1).

## Surgical Technique

### Indication and Diagnosis

**Fig 1:**
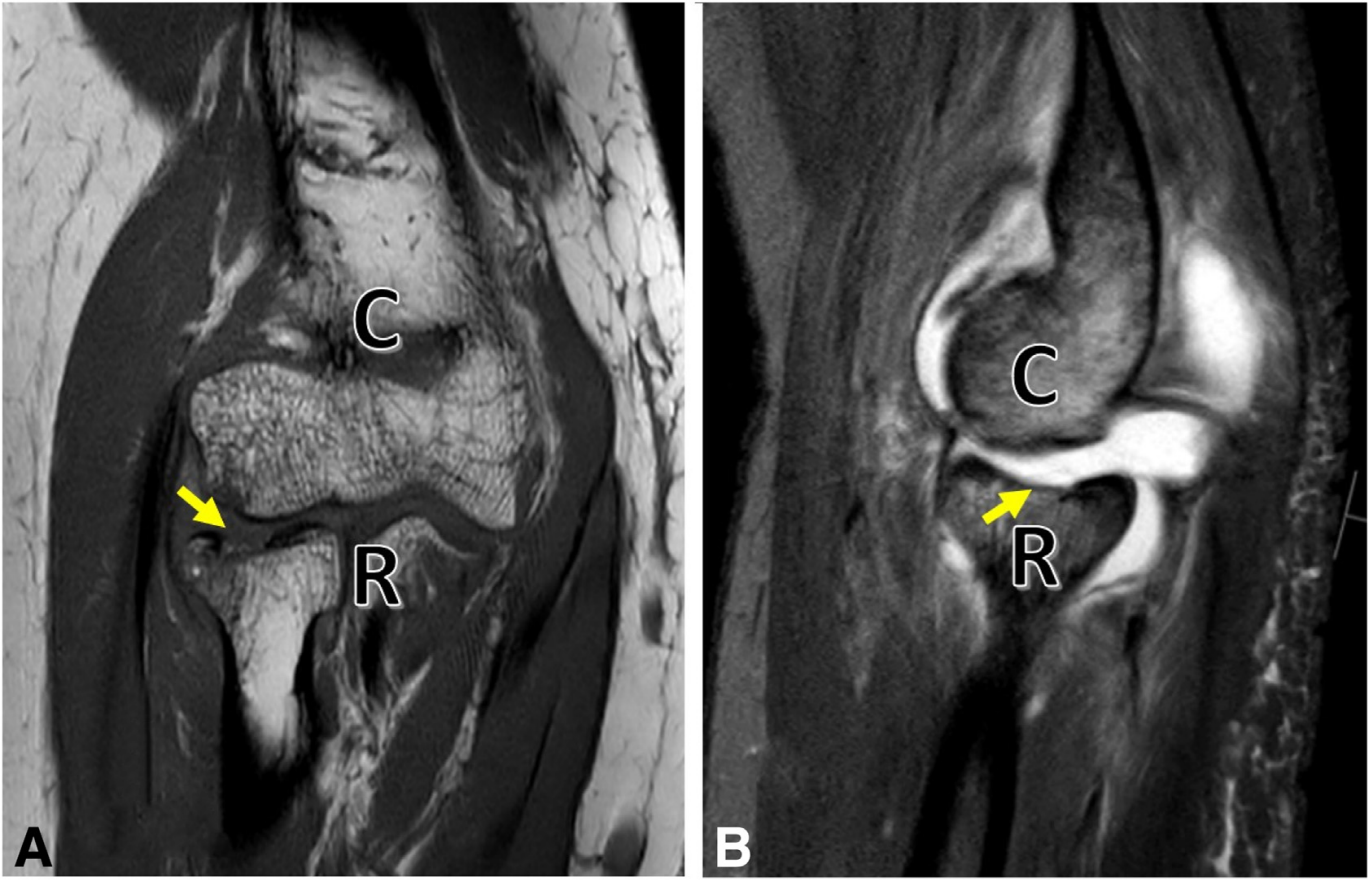
(A) Coronal and sagittal magnetic resonance imaging of the right elbow, showing the osteochondral lesion (arrow) of the radial head (R). (B) Magnetic resonance imaging of the elbow joint preoperatively with an osteochondral lesion. The present case revealed a 9-mm × 10-mm osteochondral fragment of the articular fovea of the radial head and a concomitant rupture of the lateral ulnar collateral ligament. (C, capitulum humeri.)

Surgical indication is based on clinical examination, x-ray, computed tomography, and magnetic resonance imaging. By combining computed tomography and magnetic resonance imaging, a good estimation of defect size, defect location, and fragment integrity can be achieved and is therefore recommended (Fig 1). The indication to refixate or remove an osteochondral fragment strongly depends on its size and integrity.

### Diagnostic Arthroscopy

**Fig 2:**
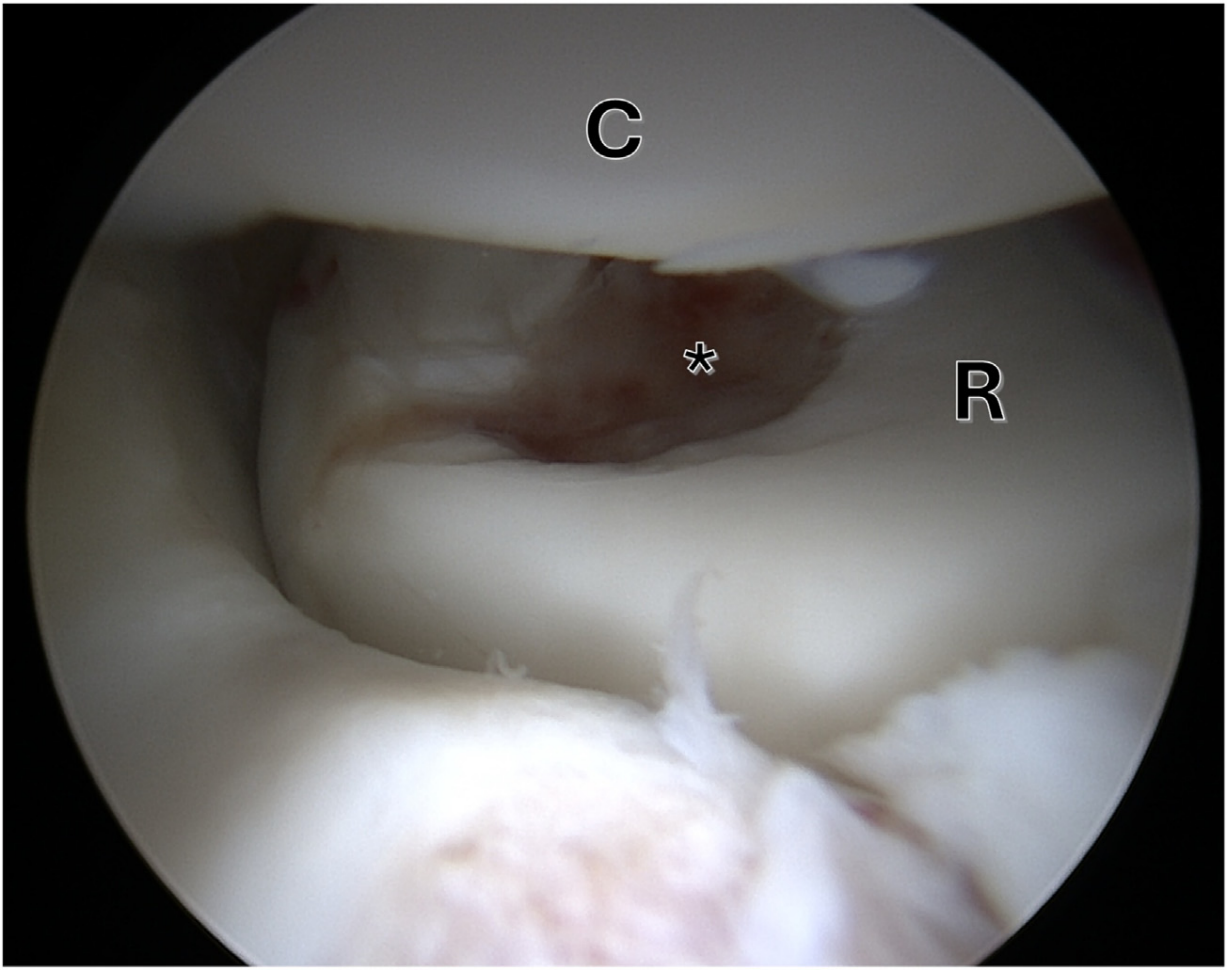
Arthroscopic view of the right elbow shows the osteochondral defect (asterisk) of the radial head (R). (C, Capitulum humeri.)

The patient is placed in a lateral position, which allows stable positioning of the affected arm and good access to all elbow joint compartments. The arm is hold by a height-adjustable padded arm bar to position the elbow joint at 90° of flexion and allowing great range of motion. A tourniquet is placed around the upper humerus. An initial diagnostic arthroscopy is recommended for joint evaluation, defect size estimation, and checking the integrity of the cartilage fragment (Fig 2). Furthermore using an arthroscopic grasper, the osteochondral fragment can be removed. However, if the size of the fragment does not allow retrieval through the arthroscopic portal, the fragment may be removed later, after arthrotomy has been performed. If the fragment is too small or broken and can therefore no longer be refixed, arthroscopic debridement or an appropriate alternative cartilage regenerative arthroscopic procedure is indicated.

### All-Suture Refixation Technique

**Fig 3:**
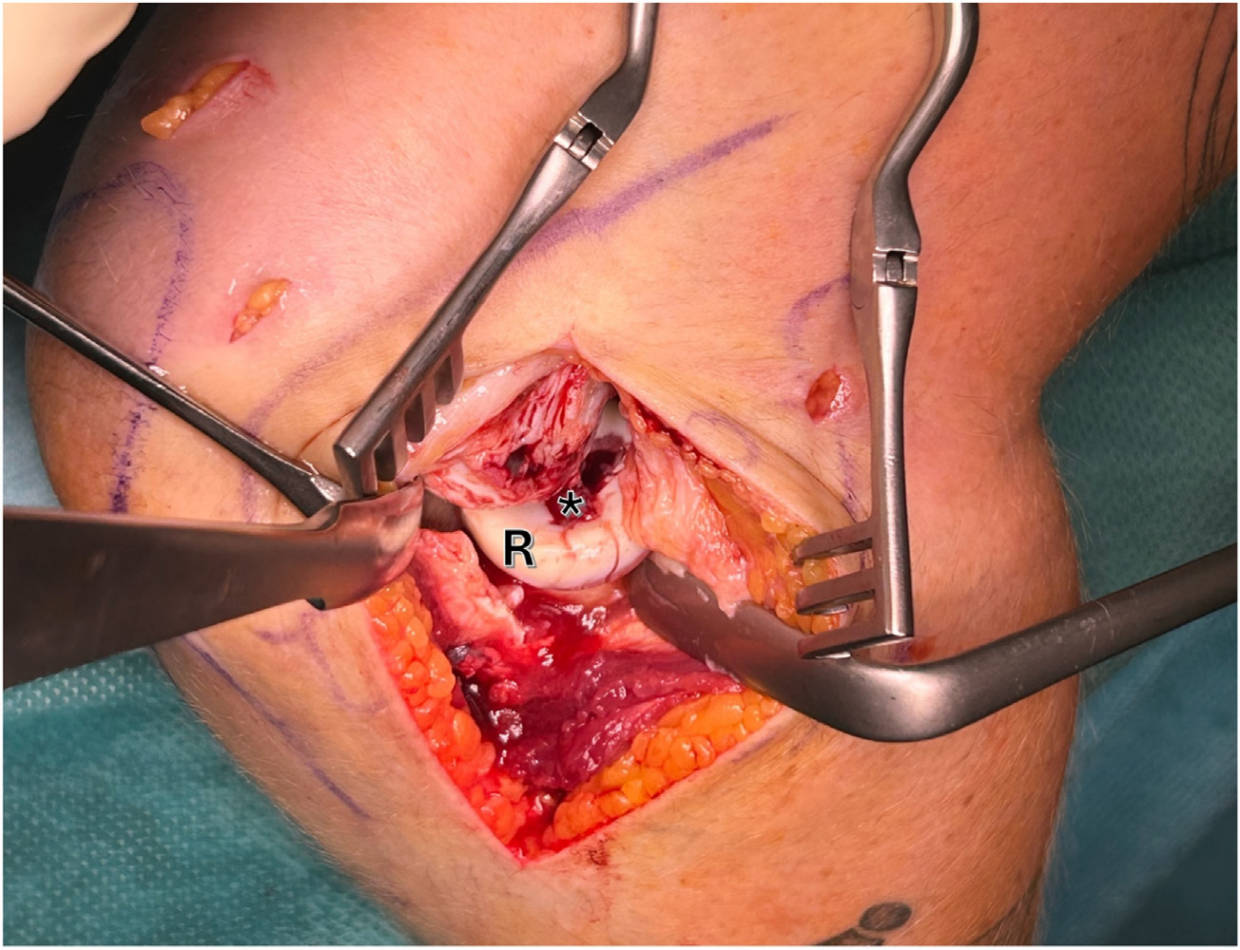
Right elbow, lateral view: an open modified Kocher approach is used to visualize the osteochondral defect (asterisk) of the radial head (R).

A lateral approach is performed. The “Kocher” interval is identified and opened between the musculus anconeus and musculus extensor carpi ulnaris. During subcutaneous preparation, utmost attention must be paid to the posterior antebrachial cutaneous nerve. The joint capsule is carefully opened, and the annular radial ligament is split to allow visualization of the entire radial head (Fig 3).

**Fig 4:**
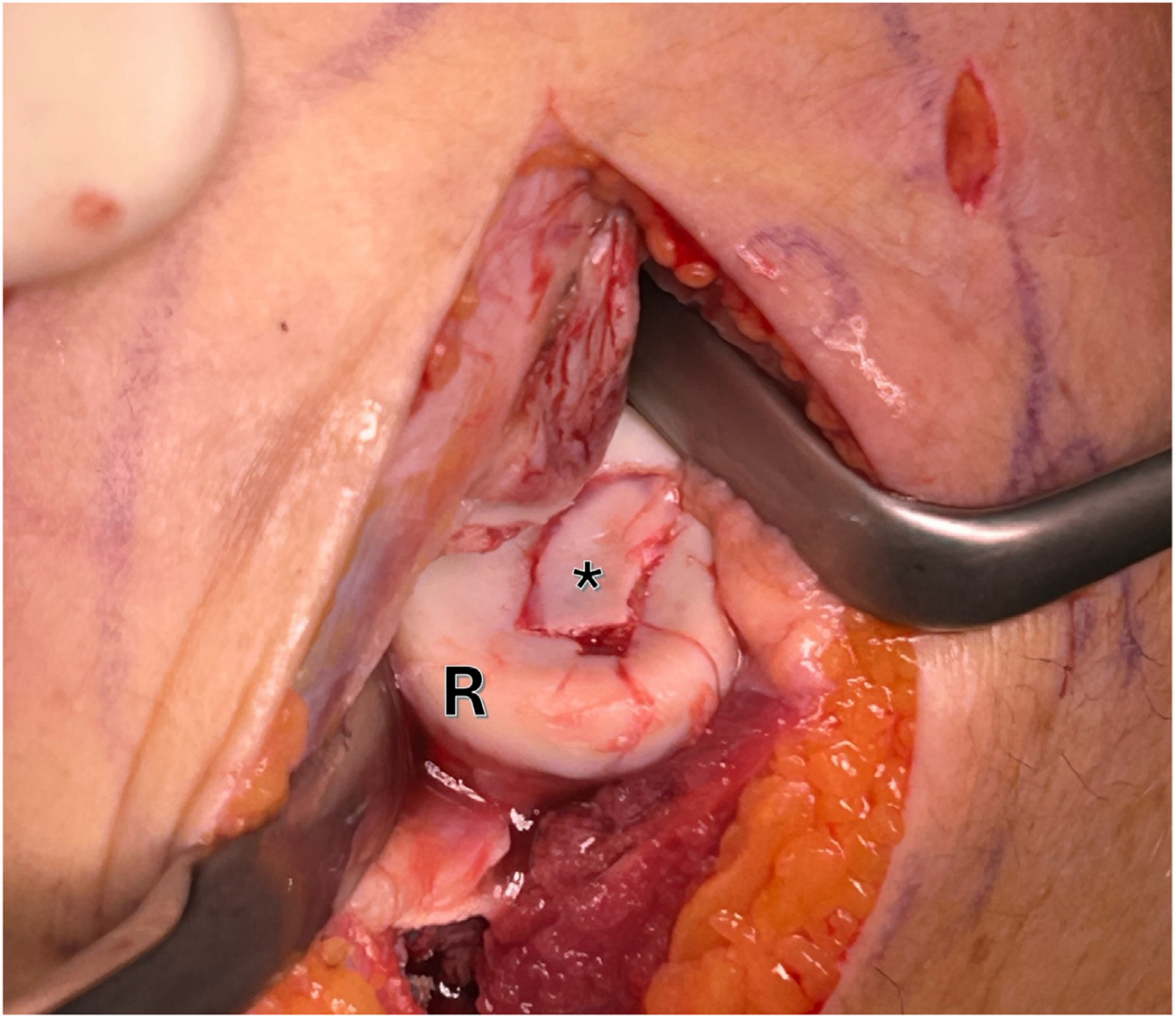
Right elbow, lateral view: the osteochondral fragment (∗) is now placed into the defect of the radial head (R). If necessary, the size needs to be adjusted using a scalpel.

Next, debridement of the defect area is necessary to create stable surroundings and a vital/bleeding base for enhanced healing of the fragment. The osteochondral fragment is now placed into position and adjusted in size using a scalpel if necessary as post-traumatic swelling of the fragment with an increase in size can occur (Fig 4).

**Fig 5:**
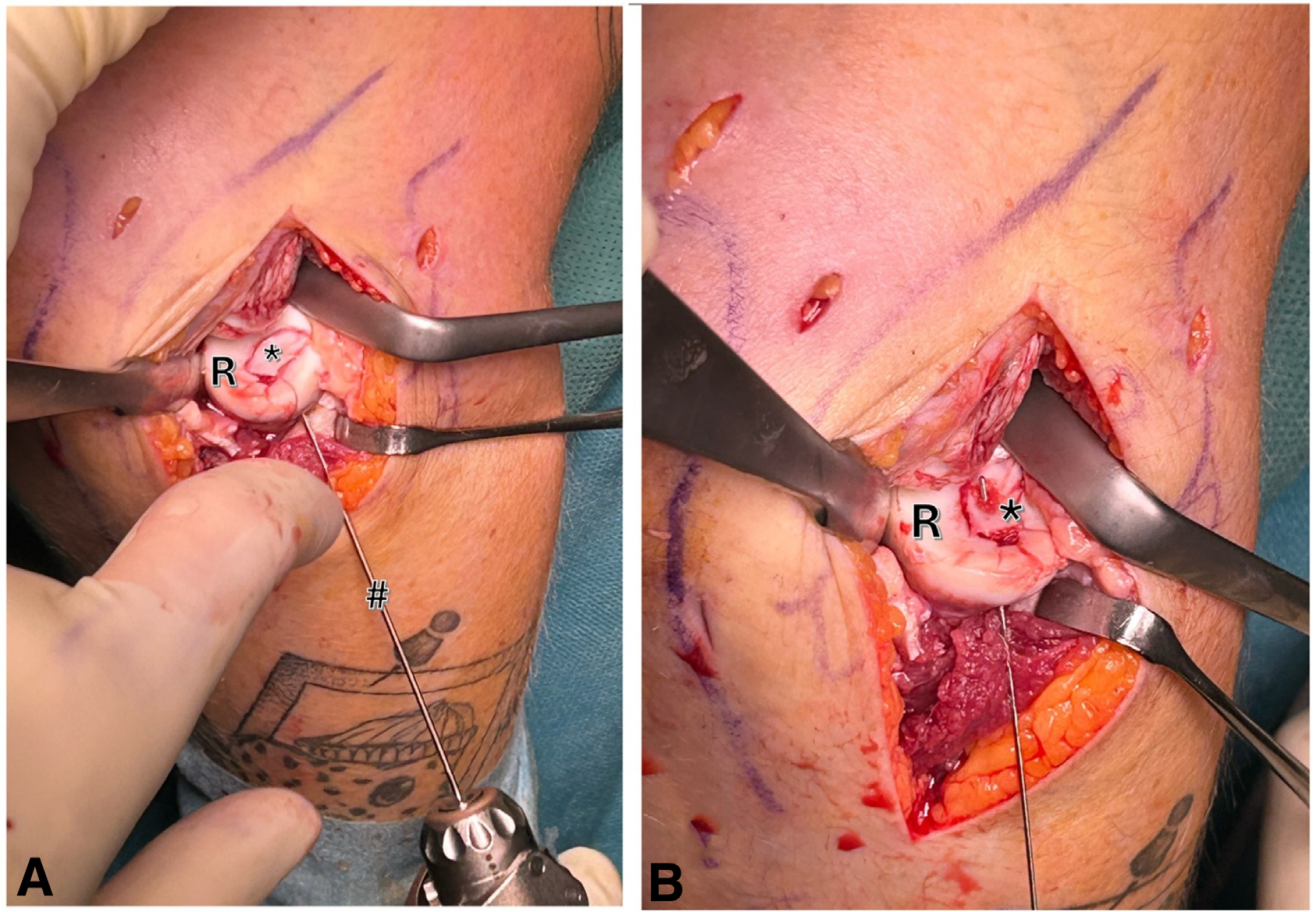
Right elbow, lateral view: using a 1.2-mm k-wire (#), 2 drill holes are placed in the fragment (asterisk) starting at the anterolateral radial neck. A bony bridge is left between the 2 drill holes in order to tie the sutures. (R, radial head.)

Subsequently, beginning at the anterolateral radial neck, two 1.2-mm drill holes are carefully placed in the fragment. A bony bridge is intentionally retained between the drill holes to facilitate secure suture tying (Fig 5 A and B).

**Fig 6:**
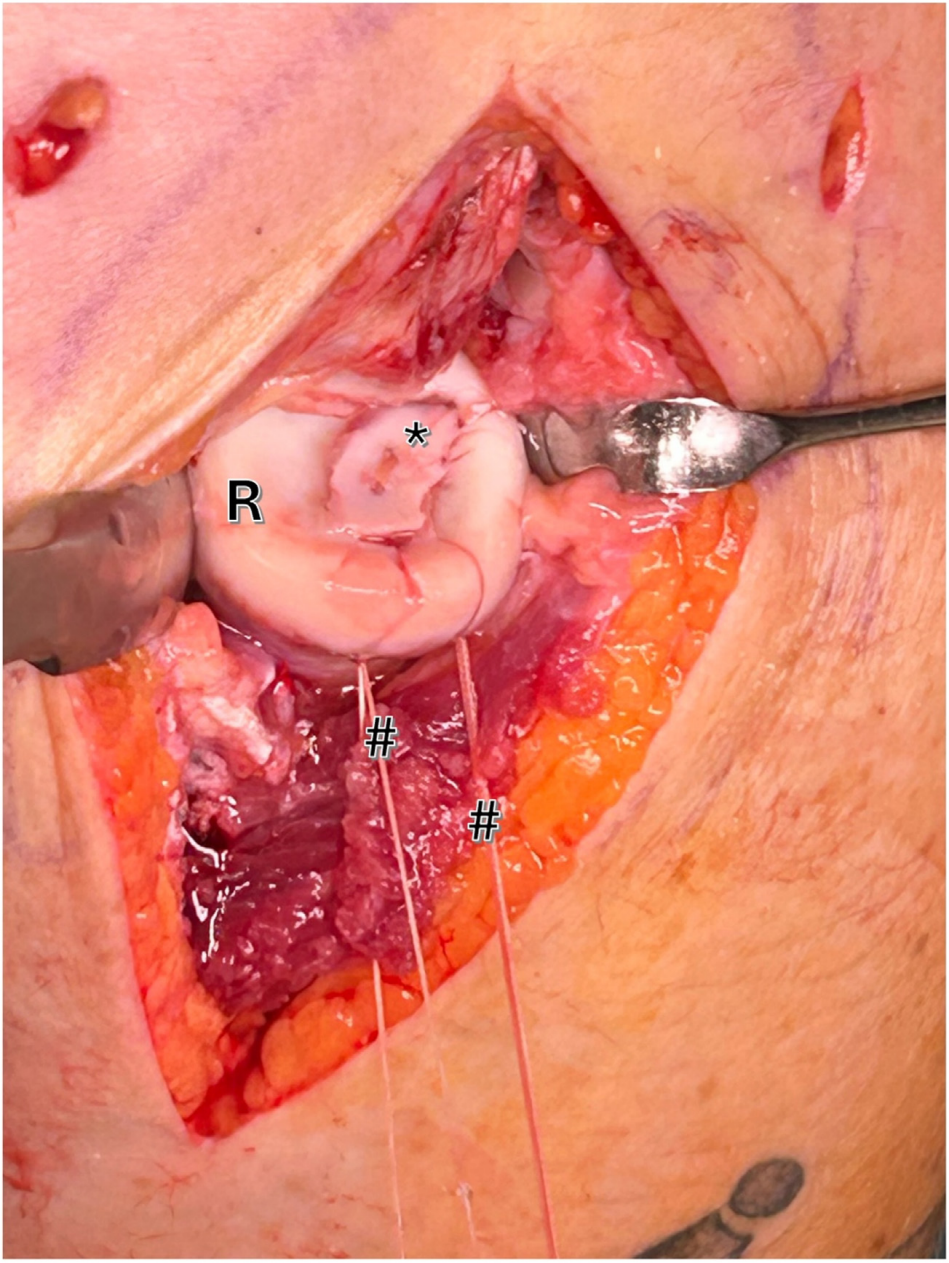
Right elbow, lateral view: utilizing a flexible loop wire, 2 No. 2 (hashtag) sutures are threaded through the drill holes in a U-shaped technique. Gentle tension on these sutures allows for precise positioning and secure fixation of the fragment (asterisk) within the defect bed of the radial head (R).

Using a flexible loop wire (e.g., Nitinol Suture Passing Wire; Arthrex), 2 resorbable No. 2 sutures (e.g., No. 2 Vicryl; Ethicon) are passed through the fragment using a U-suture technique (Fig 6). By pulling the sutures, the fragment “lands” on the defect zone like a parachute.

**Fig 7:**
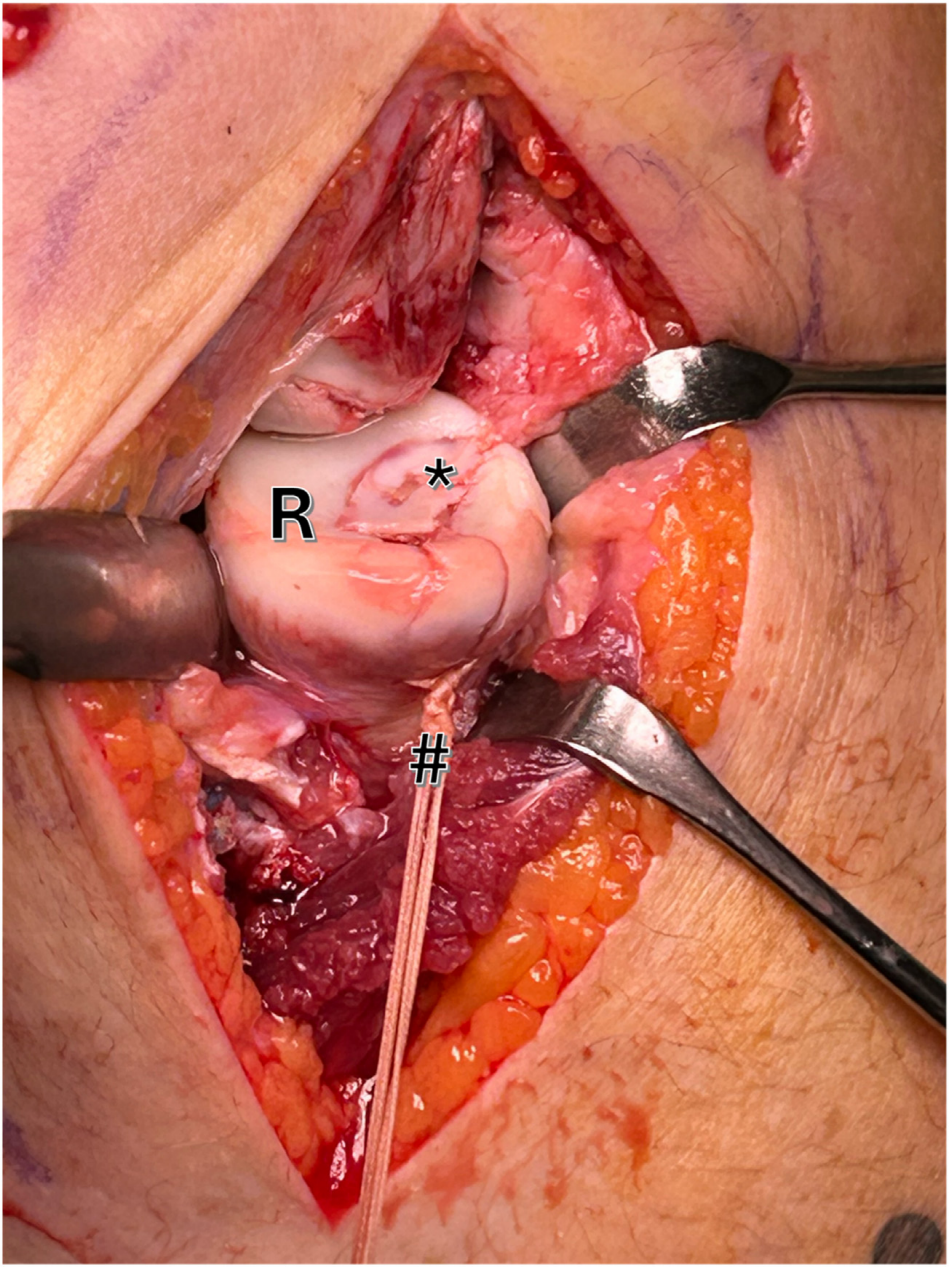
Right elbow, lateral view: the 2 No. 2 sutures are tied over the previously established bone bridge (hashtag). This method ensures a stable transosseous refixation, promoting optimal stability. Asterisks represents the osteochondral fragment (R, radial head.)

The fragment can now be securely fixed by tying both sutures over the previously established bone bridge. This method ensures a stable transosseous refixation, promoting optimal stability (Fig 7).

Following successful refixation of the osteochondral fragment, the annular ligament split and joint capsule are closed using resorbable sutures. Subsequent surgical procedures, such as the refixation or reconstruction of the lateral ulnar collateral ligament, may also be necessary.

### Postoperative Rehabilitation

Starting from the first day after surgery, early passive mobilization and exercises for the elbow joint are permitted. An elbow brace is worn for the initial 6 weeks. Range of motion is restricted to 0°, 20°, and 90° for extension and flexion during the first 2 weeks, gradually increasing to 0°, 10°, and 110° by weeks 3 and 4 and unrestricted thereafter. Pronation and supination are restricted for the first 2 weeks postoperatively. Following the removal of the brace, active range-of-motion exercises and strengthening are recommended.

## Discussion

Elbow dislocations, along with injuries to the bony and ligamentous structures of the elbow joint, are a frequently encountered injury pattern and can be challenging to manage. These dislocations often result in osteochondral fractures, creating large defect zones and increasing the risk of post-traumatic osteoarthritis. Striving for anatomic reduction and stable fixation of the fragment is ideal, yet frequently challenged by the small fragment size, in contrast to the relatively expansive options for osteosynthesis. Various techniques, using either screws or pins but also fibrin glue, have been described. Each technique offers advantages and disadvantages.

In contrast to traditional screw osteosynthesis, the current all-suture technique offers both clinical and economic advantages. By utilizing resorbable sutures, the need for a secondary surgical procedure for screw removal is eliminated. Additionally, the suture material can provide stable fragment stabilization and distribute pressure uniformly across the fragment, in contrast to the focused point pressure often associated with screw or pin fixation. This is believed to be advantageous for the healing process. While alternative fragment-preserving techniques involving fibrin glue are discussed in the literature, their limitations primarily pertain to the achieved stability.^6^ In contrast to techniques utilizing bioresorbable screws such as hydroxyapatite screws, the parachute technique offers the benefit of being achievable with standard orthopaedic surgical instruments without the need for specialized equipment, thus presenting economic advantages.^3^^,^^4^ Advantages and disadvantages of the present technique are described in Table 1.

**Table 1:** Advantages and Disadvantages

Aspect	Advantages	Disadvantages
Clinical benefits	- Preserves cartilage and bone fragment integrity - No need for implant removal	- Potential failure to adequately stabilize larger or more fragmented osteochondral defects
Economic considerations	- Avoids the need for costly specialized equipment - No need for secondary implant removal	- May still require supplementary procedures (e.g., ligament reconstruction) in complex injuries
Surgical technique	- Minimally invasive - Resorbable sutures avoid permanent implants - Easy to perform	- Suture material may not provide as rigid fixation as screws or pins for larger fragments

In summary, the technique described here is a minimally invasive, cartilage-sparing and fragment-preserving, simple and economical method for the treatment of osteochondral radial head defects. A summary of pearls and pitfalls for the clinical practice is described in Table 2.

**Table 2:** Pearls and Pitfalls

Pearls	• Stable fragment fixation: Use a U-suture technique for uniform pressure distribution across the fragment, promoting better healing. • Minimally invasive approach: The all-suture technique spares surrounding soft tissues and avoids cartilage damage, contributing to better functional outcomes. • Early rehabilitation: Allows for quicker postoperative mobilization, reducing the risk of stiffness while maintaining stability.

Pitfalls	• Fragment size limitation**:** This technique may be less effective for very large or highly fragmented osteochondral defects. • Suture tension: Proper tensioning of sutures is critical—overtightening could damage the cartilage, while undertightening could lead to insufficient stabilization. • Intraoperative visualization: Ensure adequate exposure and careful handling during the lateral approach to avoid nerve injury, particularly the posterior antebrachial cutaneous nerve.

## Disclosures

All authors (A.R., M.S.B., J.F.S.C., L.L., J.M., S.S.) declare that they have no known competing financial interests or personal relationships that could have appeared to influence the work reported in this paper.

## Supplementary Data

Video 1 Radial head osteochondral flake refixation with a transosseous all-suture technique.
